# Identification of a Novel N7-Methylguanosine-Related LncRNA Signature Predicts the Prognosis of Hepatocellular Carcinoma and Experiment Verification

**DOI:** 10.3390/curroncol30010035

**Published:** 2022-12-28

**Authors:** Chou Yang, Lingyan Zhang, Xin Hao, Mengdie Tang, Bin Zhou, Jinlin Hou

**Affiliations:** State Key Laboratory of Organ Failure Research, Guangdong Key Laboratory of Viral Hepatitis Research, Department of Infectious Diseases and Hepatology Unit, Nanfang Hospital, Southern Medical University, Guangzhou 510515, China

**Keywords:** N7-methylguanosine, lncRNA, hepatocellular carcinoma, prognostic signature, NRAV

## Abstract

(1) Background: It is well-known that long non-coding RNAs (lncRNAs) and N7-methylguanosine (m7G) contribute to hepatocellular carcinoma (HCC) progression. However, it remains unclear whether lncRNAs regulating m7G modification could predict HCC prognosis. Thus, we sought to explore the prognostic implications of m7G-related lncRNAs in HCC patients. (2) Methods: Prognostic M7G-related lncRNAs obtained from The Cancer Genome Atlas (TCGA) database were screened by co-expression analysis and univariate Cox regression analysis. Next, the m7G-related lncRNA signature (m7GRLSig) was conducted by Least absolute shrinkage and selection operator (LASSO) Cox regression and multivariate Cox regression analysis. Kaplan–Meier analysis and time-dependent receiver operating characteristics (ROC) assessed the prognostic abilities of our signature. Univariate and multivariate Cox regression, nomogram, and principal component analysis (PCA) were conducted to evaluate our signature. Subsequently, we investigated the role of m7GRLSig on the immune landscape and sensitivity to drugs in HCC patients. The potential function of lncRNAs obtained from the prognostic signature was explored by in vitro experiments. (3) Results: A novel m7GRLSig was identified using seven meaningful lncRNA (ZFPM2-AS1, AC092171.2, PIK3CD-AS2, NRAV, CASC19, HPN-AS1, AC022613.1). The m7GLPSig exhibited worse survival in the high-risk group and served as an independent prognostic factor. The m7GRLSig stratification was sensitive in assessing the immune landscape and sensitivity to drugs between the high-risk and low-risk groups. Finally, in vitro experiments confirmed that the knockdown of NRAV was accompanied by the downregulation of METTL1 during HCC progression. (4) Conclusions: The m7G-related signature is a potential predictor of HCC prognosis and contributes to individualize the effective drug treatment of HCC.

## 1. Introduction

Primary liver cancer (PLC) is ranked sixth for incidence of cancer and third for cancer death [1]. Hepatocellular carcinoma (HCC) arises after the occurrence of various chronic liver diseases and accounts for 90% of PLC [2,3]. Currently, there are no effective curative treatments for early-stage HCC other than hepatectomy or liver transplantation. Chemoembolization, radiofrequency ablation, and systemic therapies remain the primary options for advanced HCC patients who are ineligible for surgery [4,5]. The overall response to these therapies remains poor due to de novo recurrence and frequent metastasis [6]. Owing to the extensive heterogeneity and diversity of HCC, a new and better diagnostic approach is urgently needed to improve individualized diagnosis and treatment.

The development of cancer is often accompanied by genetic alterations and dysregulation of epigenetics [7]. Abnormal epigenetic modifications interfering with RNA may lead to cancer initiation and progression [8]. Various RNA types in cells undergo posttranscriptional modifications that affect RNA metabolism and the regulation of gene expression [9]. N7-methylguanosine (m7G) is generated by the methylation of guanine at the seventh position as a unique post-transcriptional regulator [10]. It mediates RNA metabolism and protein translation during the RNA life cycle, including transcriptional elongation, pre-mRNA splicing, and mRNA translation [11,12,13]. The m7G modification has been confirmed to be regulated by many genes. Methyltransferase-like 1 (METTL1) catalyzes the m7G modification at the target mRNA via the WD repeat domain 4 (WDR4) [14,15]. RNA methyltransferase-RNA-activating miniprotein (RNMT-RAM) produces an m7G cap to regulate mRNA processing, export, and translation [16]. Interferon-induced proteins with tetratricopeptide repeats 5 (IFIT5) recognizes the triphosphate bridge linking the m7G cap and then reduces the 48S translational initiation complex [17]. The human decapping mRNA 2 (DCP2) protein contains a highly conserved motif that can hydrolyze m7G-cap [18]. The m7G modification has been implicated in regulating cancer progression in various ways [19,20,21,22]. The m7G modification of tRNA mediated by METTL1/WDR4 drives esophageal squamous cell carcinoma progression [19]. METTL1 promotes bladder cancer progression by modulating m7G tRNA modification of EGFR/EFEMP1 [20]. METTL1-mediated m7G tRNA modification promotes HCC cell growth and behavior by inhibiting PTEN-related signaling pathways [21]. METTL1 promotes lung cancer progression by affecting the AKT/mTORC1 signaling pathway [22].

Long non-coding RNAs (lncRNAs) are recognized as nonprotein-coding RNAs containing >200 bases [23]. lncRNAs affect diverse physiological processes by affecting protein or nucleic acid metabolism and epigenetic modifications [24]. Accumulating evidence has confirmed that aberrant lncRNA expression drives the occurrence, development, and metastasis of HCC [25,26]. Therefore, we believe that m7G-related lncRNAs are involved in HCC development. However, the prognostic value of lncRNAs regulating m7G modification in HCC is unclear and remains to be further explored.

Therefore, we generated a novel m7G related-lncRNAs prognostic signature to stratify HCC patients for their prognosis and validated its predictive value for prognosis. The associations between a prognostic signature and clinical features, m7G-related biological pathways, immune landscape, and sensitivity to clinical drug treatment for patients with HCC were explored using the relevant public data. Finally, in vitro experiments explored the expression and function of lncRNAs obtained from the prognostic signature.

In summary, the m7G-related prognostic signature exhibited a great ability to predict HCC patients’ survival and drug sensitivity and describe the immune landscape of HCC patients.

## 2. Materials and Methods

### 2.1. Patients and Datasets

**Figure 1:**
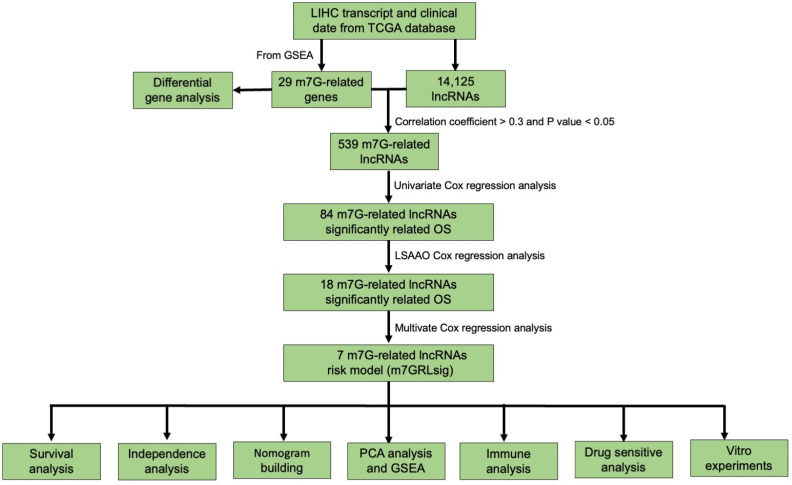
Workflow of this study. TCGA, The Cancer Genome Atlas; LIHC, hepatocellular carcinoma; m7G, N7-methylguanosine; lncRNAs, long no-coding RNAs; OS, overall survival; PCA, Principal component analysis; GSEA, gene set enrichment analysis.

Figure 1 showed the workflow of this study. The original transcriptome sequencing data and HCC clinical information were downloaded from The Cancer Genome Atlas (TCGA) website (https://portal.gdc.cancer.gov/ accessed on 6 May 2022). Patients with complete clinical information were screened for further analyses according to the criteria of a follow-up greater than 30 days. The features of the samples are summarized in Supplementary Table S1.

### 2.2. M7G-Related Genes Selection

The 29 m7G-related genes were obtained from the literature [27] and the Gene Set Enrichment Analysis (GSEA) (http://www.gsea-msigdb.org/gsea/index.jsp, accessed on 6 May 2022), including gene sets: GOMF_RNA_CAP_BINDING.gmt, GOMF_RNA_7_METHYLGUANOSINE_CAP_BINDING.gmt, GOMF_m7G_5_PPPN_DIPHOSPHATASE_ACTIVITY.gmt.

### 2.3. Establishment of N7-Methylguanosine-Related lncRNA Signature (m7GRLsig)

M7G-related lncRNAs were identified by the co-expression analysis of m7G-related messenger RNA (mRNA) and lncRNA based on the following criteria: |coefficient| > 0.3 and *p* < 0.001 using the “limma” R package. Significant prognostic m7G-related lncRNAs screened by univariate Cox regression analysis (*p* < 0.05) were subjected to the least absolute shrinkage selection operator (LASSO) regression. Based on the results of LASSO regression analysis, multivariate Cox regression analysis was further used to screen m7G-related lncRNAs to establish a prognostic signature. The equation for calculating the risk score is as follows:

$$\overset{n}{\underset{i}{\sum }}\left(Coef*\exp\right)lncRNAi$$

the coefficient value was abbreviated as *coef* and lncRNA expression was abbreviated as exp. We dichotomized HCC patients into low- and high-risk groups using the cutoff of the median risk score and randomly partitioned them into two internal sets.

### 2.4. Nomogram Construction

A nomogram was formulated using the “rms (4.0.3)” package. The agreement between the practical and predicted outcomes was evaluated by the calibration curve.

### 2.5. Immune Landscape Investigation

The immunocytes infiltration proportion was explored using CIBERSORT (http://cibersort.stanford.edu/, accessed on 7 May 2022). The immune checkpoint genes expression, and immunocytes infiltration fraction/function were evaluated using the “GSVA”, “ggpubr”, and “limma” R packages. All results were visualized by “barplot”, “corrplot”, and “ggplot2” R package.

### 2.6. Drug Sensitivity Analyses

The half-maximal inhibitory concentration (IC50) obtained from the Genomics of Drug Sensitivity in Cancer database (https://www.cancerrxgene.org, accessed on 7 May 2022) was used to assess the potential ability of the risk mod of m7GRLSig in predicting the sensitivity to the antitumor drug for HCC using the “pRRophetic” R package [28].

### 2.7. Cell Culture

We purchased Huh7 from the Cell Resource Center of the Chinese Academy of Sciences (Shanghai, China). The normal liver cell line (L02) and HCC cell lines (HepG2, SMMC-7721, MHHC-97H) were maintained in our lab. Cells were propagated in DMEM (Gibco) 10% fetal bovine serum (FBS; Gibco) and 1% penicillin and streptomycin (Gibco) under 5% CO_2_ at 37 °C.

### 2.8. PCR for Quantitative Reverse Transcription (qRT-PCR)

The total RNA of cell lines was extracted by RNA purification kit (EZBioscience, the United States according to the protocol and measured for concentration and purity using a Nano drop2000 (Thermo). The expression of mRNAs was detected using the SYBR Green (Takara, Beijing, China) on Lightcycler 480 II (Roche). Primers and their sequences were listed in Supplementary Table S2. The comparative Cp value of the GAPDH gene normalized the expression of mRNA.

### 2.9. Cell Transfection

Before transfection, cells were added to a six-well plate (3 × 10^5^/well). Si- negative control (si-NC) and si-NRAV were chemically synthesized by RiboBio (Guangzhou, China). Lipofectamine RNAIMAX (Invitrogen, Waltham, MA, USA) was utilized to transfect si-NRAV and si-NC into Huh7 and HepG2 cells. Si-NRAV sense 5′-GGATGGATAGTTCAGAGTA-3ʹ.

### 2.10. Proliferation Assay

Huh7 cells (1 × 10^3^/well) and HepG2 cells (1 × 10^3^/well) were added to 96-well plates after 48 h post-transfection with si-NRAV or si-NC and 10 μL of cell counting Kit-8 (CCK8) solution was applied per well at 24, 48, 72, 96 h. After 2 h incubation, the optical density (OD) values were measured by a microplate reader (Synergy HT) at 450 nm. Huh7 cells (1 × 10^3^/well) and HepG2 cells (1 × 10^3^/well) were added to a 6-well plate after 48 h of cell transfection, and then cultured for 2 weeks for colony formation assays. Next, the colonies were fixed in 4% paraformaldehyde (Solarbio, Beijing, China) for 30 min followed by 1% crystal violet (Solarbio) staining. The colony numbers were calculated by ImageJ software.

### 2.11. Transwell Analysis

A total of 6 × 10^4^ transfected Huh7 cells or HepG2 cells were added in the upper chamber (pore 8.0 μm pore; BD FALCON) containing 200 μL DMEM (serum-free) with or without Matrigel (BD Biosciences), whereas the bottom compartment was filled with 600 μL DMEM (10% FBS). Following 36 h incubation at 37 °C, unmigrated cells were removed from the upper chamber and fixed in 4% paraformaldehyde (Solarbio) for 30 min followed by 1% crystal violet (Solarbio) staining. Migrating and invading cells were captured under an inverted microscope (Olympus, Tokyo, Japan) and counted in three random fields using ImageJ software.

### 2.12. Statistical Analysis

The “ggalluvial” R package and Cytoscape 3.7.2 were used to construct the Sankey diagrama of the lncRNA-mRNA co-expression network. The overall survival (OS) was compared using Kaplan–Meier (KM) curves and the log-rank test. The “scatterpolt3D” R package performed principal component analysis (PCA). The functional annotation of these selected lncRNAs was explored by means of GSEA. The R (version 4.1.0) software conducted all statistical analyses. Differences between the NRAV knockdown-treated cells and NC-treated cells were compared by Student’s *t*-test. Values of *p* < 0.05 were defined as the significance threshold.

## 3. Results

### 3.1. Differentially Expressed m7G-Related Genes in HCC

**Figure 2:**
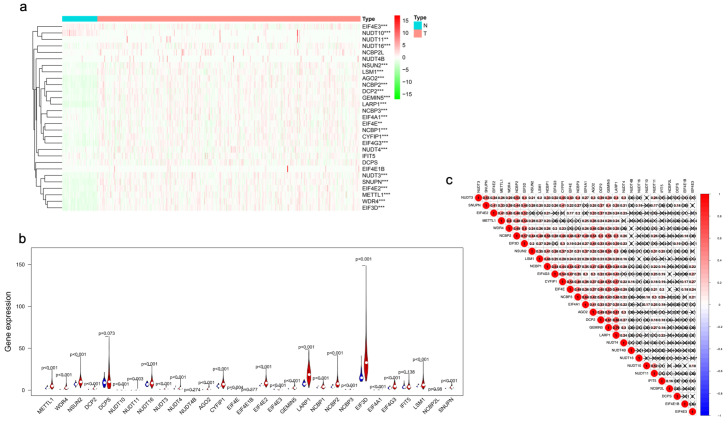
Expression pattern of m7G-related genes in HCC. (**a**) The heatmap visualizes the expression levels of m7G-related gene in each sample. Green represents low expression and red represent high expression. (**b**) The vioplot shows the differentially m7G-related gene in HCC. Blue represents normal sample and red represents HCC sample. White spot represents the median value of expression. (**c**) Spearman correlation analysis of the 29 m7G-related gene in HCC. HCC, hepatocellular carcinoma; m7G, N7-Methylguanosine; N, normal samples; T, tumor samples. ** *p* < 0.01; *** *p* < 0.001.

We performed differential expression analysis for 29 m7G-related genes based on the expression data in TCGA-LIHC (371 HCC samples and 50 normal samples). The differential expression pattern was presented as a heatmap (Figure 2a). Specifically, NUDT11 expression levels were remarkably higher while NUDT10 had remarkably lower levels in tumor tissues (Figure 2b, Supplementary Table S3). Furthermore, correlation analysis showed intrinsic associations among the 29 m7G-related genes (Figure 2c).

### 3.2. Identification of the m7G-Related LncRNA Predictive Signature

**Figure 3:**
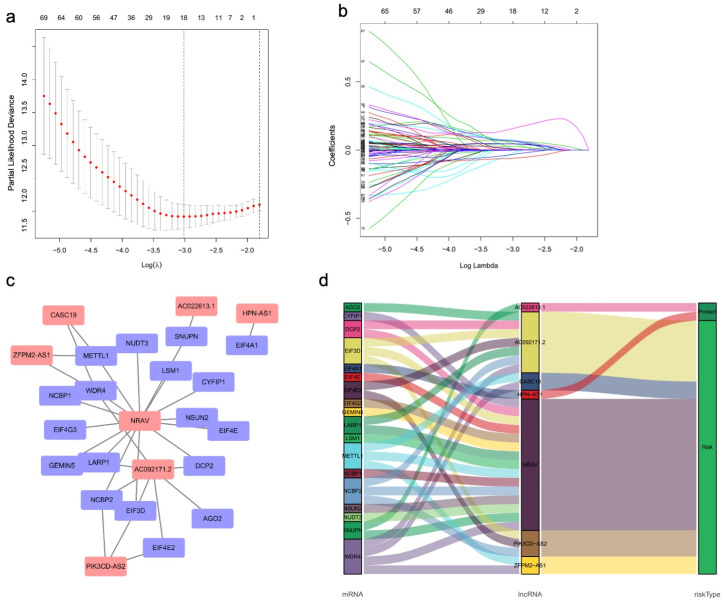
Screened m7G-related lncRNAs with significant prognostic value in HCC. (**a**) LASSO Cox regression using 10-fold cross-validation was used to determine the optimal factors for select independent factors. The vertical dashed lines represent the optimal parameter (lambda) value. (**b**) Profiles of Lasso coefficients. (**c**) The co-expression network of prognostic m7G-related lncRNAs-mRNAs. (**d**) The Sankey diagram of the relationship between prognostic m7G-related lncRNAs, mRNAs, and risk type. HCC, hepatocellular carcinoma; m7G, N7-Methylguanosine; lncRNAs, long noncoding RNAs; LASSO, least absolute shrinkage and selection operator.

First, we screened 539 m7G-related lncRNAs by the co-expression of m7G-related mRNAs and lncRNAs using Spearman correlation analysis (|coefficient| > 0.3 and *p* < 0.001). Then, a total of 84 lncRNAs were identified as prognostic factors using univariate Cox regression (Supplementary Table S4). Lasso regression analysis was performed on these prognostic lncRNAs to avoid possible overfitting and improve prediction accuracy followed by multivariate Cox regression (Figure 3a,b). Finally, we screened seven meaningful m7G-related lncRNAs for the predictive signature (Table 1, Supplementary Figure S1). The co-expression network containing m7G-related lncRNAs-mRNAs was visualized in Figure 3c,d. The m7GRLsig-score for each patient was calculated as the flowing equation: risk score = (0.18133 × ZFPM2-AS1) + (0.36071 × AC092171.2) + (0.34046 × PIK3CD-AS2) − (0.42774 × HPN-AS1) -(0.41772 × AC022613.1) + (0.48860 × NRAV) + (0.34254 × CASC19).

### 3.3. The Prognostic Influence of m7GRLSig

**Figure 4:**
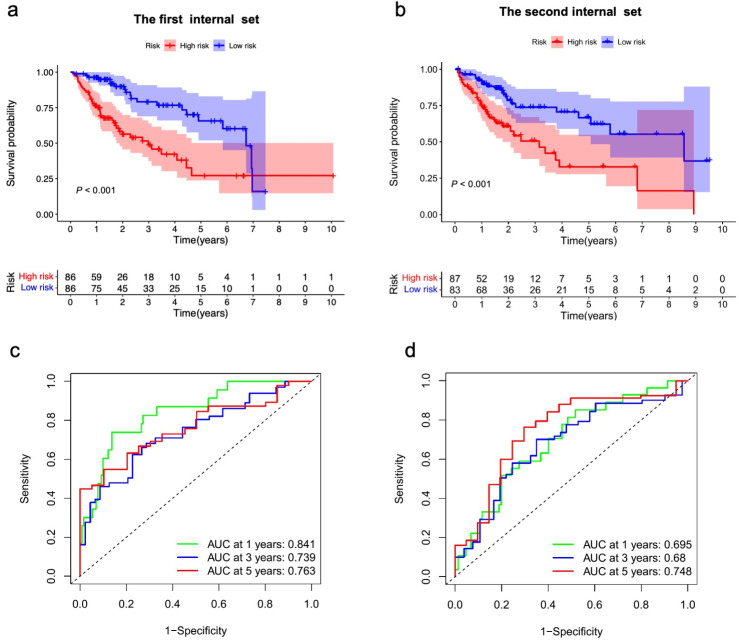
Verification of m7G-related lncRNAs prognostic signature’s prediction ability. (**a**,**b**) Kaplan–Meier survival analysis for the OS curves of HCC in the first internal set (**a**), the second internal set (**b**) between low- and high-risk groups. (**c**,**d**) The ROC analysis of the m7G-related lncRNAs risk score in the first internal set (**c**) and the second internal set (**d**). HCC, hepatocellular carcinoma; m7G, N7-Methylguanosine; lncRNAs, long noncoding RNAs; OS, overall survival.

**Figure 5:**
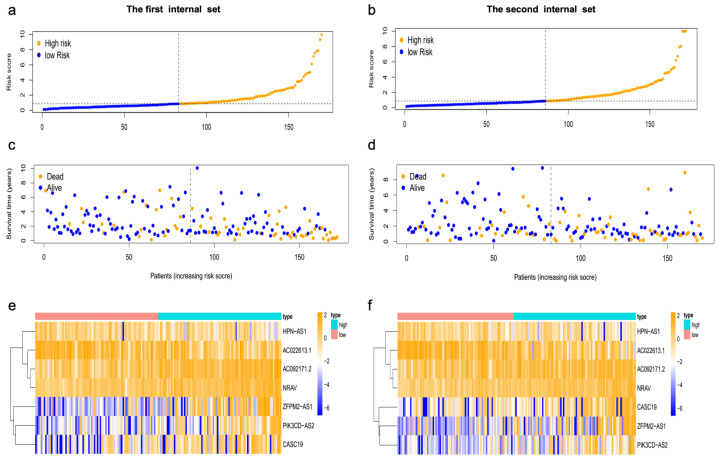
M7G-related lncRNA signature risk score analysis. (**a**,**b**) The risk score distribution in the first internal set (**a**) and the second internal set (**b**) between low- and high-risk groups. (**c**,**d**) Survival time of patients with different risk scores in the first internal set (**c**) and the second internal set (**d**). The green and orange dots represent survival and death, respectively. (**e**,**f**) The heatmap showed the expression levels of m7G-related lncRNAs the first internal set (**e**) and the second internal set (**f**) between low- and high-risk groups. m7G, N7-Methylguanosine; lncRNAs, long noncoding RNAs.

**Figure 6:**
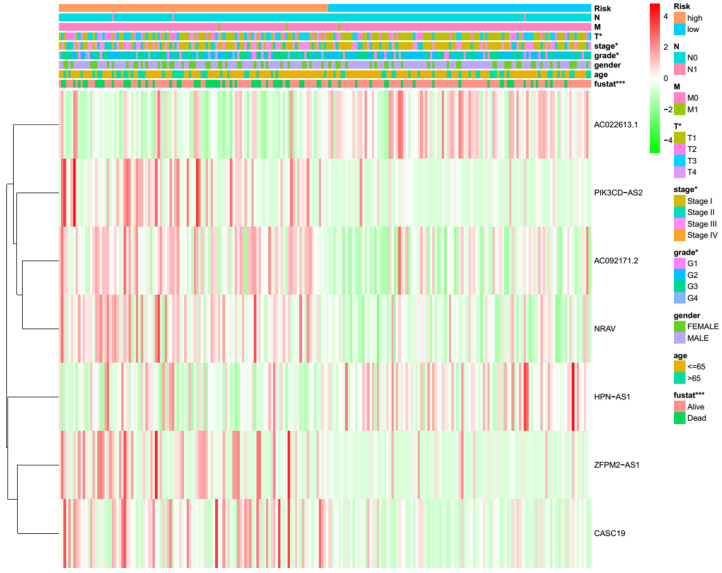
The heatmap of the distribution of 7 prognostic m7G-related lncRNAs and clinicopathological features in the high-risk and low-risk groups. m7G, N7-Methylguanosine; lncRNAs, long noncoding RNAs; T, tumor size; N, lymph node metastasis; M, distant metastasis. *, *p* < 0.05; ***, *p* < 0.001.

HCC patients were subsequently divided into low-risk and high-risk groups based on the median risk score as the cutoff and randomly assigned into two internal validation sets. Kaplan–Meier analysis showed the high-risk group had shorter OS than the low-risk group in both internal sets (all *p* < 0.001) (Figure 4a,b). The entire set yielded similar results (*p* < 0.001) (Supplementary Figure S2a). The area under the curve (AUC) values for 1-, 3-, and 5-year survival of the first internal set were 0.841, 0.739, and 0.763, respectively (Figure 4c). The AUC values for 1-, 3-, and 5-year survival of the second internal set were 0.695, 0.680, and 0.748, respectively (Figure 4d). The entire set also showed the good predictive ability of this model (Supplementary Figure S2b). The risk curve and scatterplot indicated that the survival status depended on the newly developed signature (Figure 5a–d), Supplementary Figure S2c,d). The heatmaps showed the different expression patterns of the seven lncRNAs between high- and low-risk groups (Figure 5e,f, Supplementary Figure S2e). In addition, we found the differences in T stage, stage, grade, and fustat (all *p* < 0.05) between the high- and low-risk groups (Figure 6). We assessed the OS in the subgroups with different clinicopathological features (Supplementary Figure S3). We found the m7GRLsig performed well in all the subgroups stratified by male patients (Supplementary Figure S3a) and age (<65 years, Supplementary Figure S3c), T stage (T1 and T2–4, Supplementary Figure S3e,f), stage (stage I and stage II–III, Supplementary Figure S3g,h), and grade (grade 2 and grade 3–4, Supplementary Figure S3j,k).

### 3.4. Independent Prognostic Analysis of m7GRLSig

**Figure 7:**
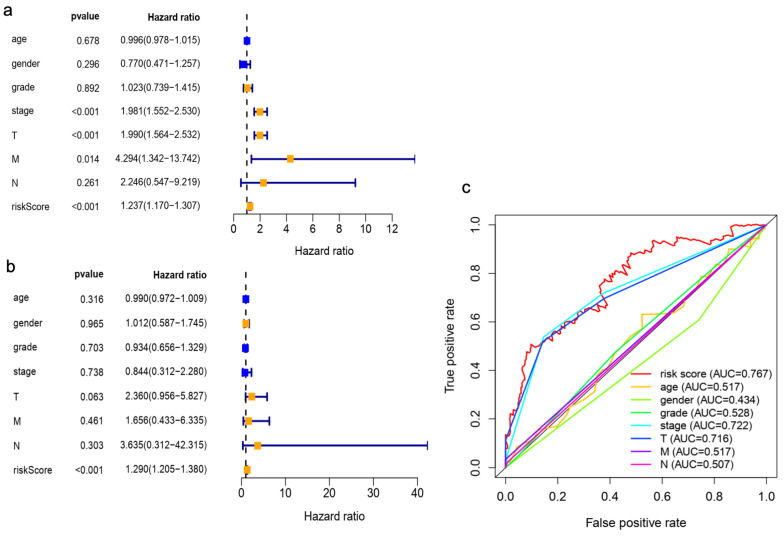
Univariate and multivariate Cox regression analysis of risk model score for HCC. Univariate (**a**) and multivariate (**b**) Cox Forest plot was performed on the risk score and clinical features. (**c**) The AUC for risk score and clinical factors and the ROC curves. HCC, hepatocellular carcinoma; T, tumor size; N, lymph node metastasis; M, distant metastasis.

**Figure 8:**
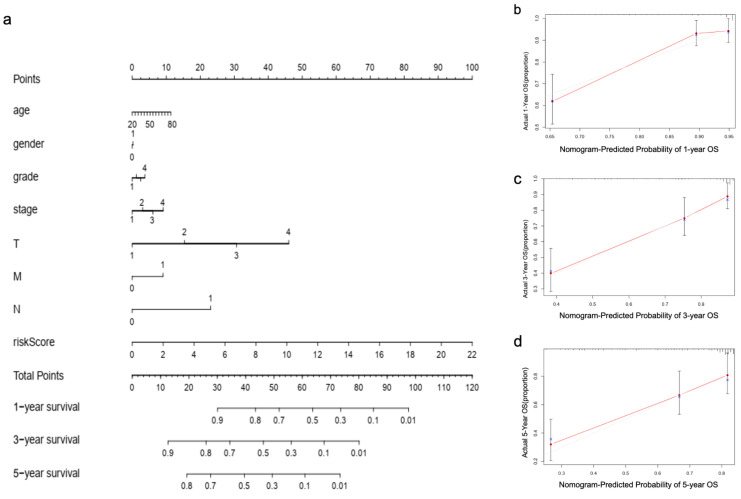
Clinical prognostic nomogram for survival prediction. (**a**) The nomogram of risk score and clinical factors predicts 1-, 3-, and 5-year of OS of HCC patients. The 1-year (**b**), 3-year (**c**), and 5-year (**d**) OS calibration curves evaluated the agreement between the predicted and the actual OS. OS, overall survival; HCC, hepatocellular carcinoma.

Univariate Cox regression analysis showed the risk score was significantly associated with the OS in HCC patients (Figure 7a). Multivariate regression analysis further indicated that risk score could serve as an independent prognostic indicator for HCC patients (Figure 7b). The AUC value of the risk score was superior to the predictive accuracy of clinical features for HCC prognosis (Figure 7c), suggesting that the risk model of M7RLSig for HCC prognosis was considered reliable. Moreover, we established a nomogram plot to predict the HCC prognosis at 1, 3, and 5 years (Figure 8a). The calibration curve showed a favorable predictive ability of the nomogram (Figure 8b–d).

### 3.5. Differential m7G Modification Statuses Stratified by m7GRLSig

**Figure 9:**
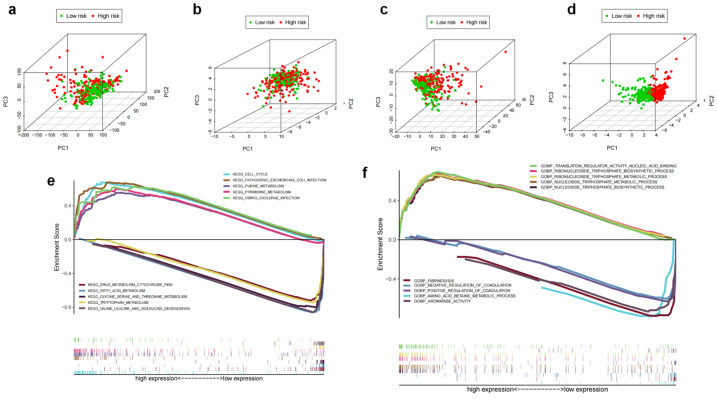
Differential analysis of m7G modification statuses between high-risk and low-risk groups. Principal component analysis (PCA) between low-risk and high-risk groups based on the whole-genome (**a**), m7G-related genes (**b**), m7G-related lcnRNAs (**c**), and the model of m7GRLSig (**d**). High-risk groups are indicated in red, and low-risk groups are indicated in green. (**e**,**f**) GSEA based on the lncRNAs of m7GRLSig. KEGG identifies high and low-risk related signaling pathways in HCC (**e**); GSEA GO identifies high and low-risk related signaling pathways in HCC (**f**). GSEA, Gene Set Enrichment Analysis; KEGG, Kyoto Encyclopedia of Genes and Genomes; GO, Gene Ontology; HCC, hepatocellular carcinoma; m7G, N7-Methylguanosine.

PCA analysis based on the whole genome, m7G-related genes, m7G-related lncRNAs, and m7GRLSig models was performed to further evaluate the m7GRLSig. The expression of the whole genome (Figure 9a), m7G-related genes (Figure 9b), and m7G-related lncRNAs (Figure 9c) could not effectively discriminate the low-and high-risk patients; however, the terms of the m7GRLSig model can effectively distinguish these two groups (Figure 9d), revealing the differential m7G modification statuses between the low- and high-risk group. The discriminative power of m7GRLSig further demonstrated this model had reliable clustering ability. The differential biological pathways associated with m7GRLSig were identified using GSEA. We found that cell cycle, purine and pyrimidine metabolism pathways were enriched in the high-risk group (Figure 9e). In addition, the metabolic and biosynthetic process of nucleoside/ribonucleoside triphosphate, translation regulator activity and nucleic acid binding were significantly enriched in the HCC patients with high-risk scores (Figure 9f). Functional annotation further confirmed that the model of m7GRLSig may be related to nucleic acid metabolism and genetic modification.

### 3.6. Correlations between Immune Landscape and m7GRLSig

**Figure 10:**
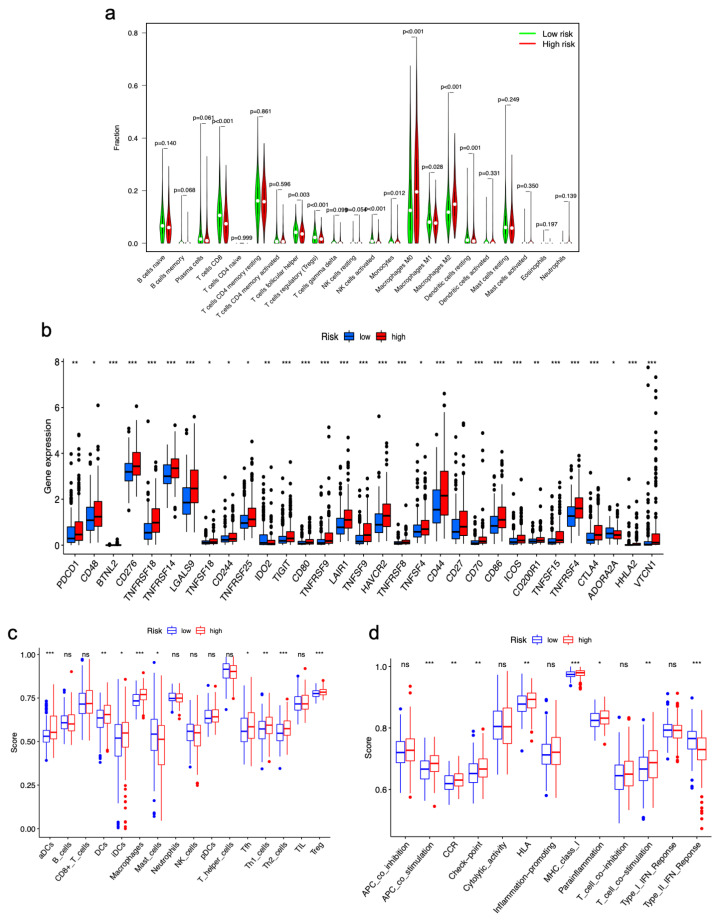
Differential immune landscape between low- and high-risk groups. (**a**) Comparison of the tumor immune cell infiltration between high- and low-risk groups. (**b**) The difference of 31 checkpoints expression in high- and low-risk groups. Comparison of the ssGSEA scores of immune cells (**c**) and immune functions (**d**) between high- and low-risk groups. ssGSEA, single sample Gene Set Enrichment Analysis. ns, no significance; *, *p* < 0.05; **, *p* < 0.01; ***, *p* < 0.001.

We investigated whether the m7GRLSig was related to the immune landscape of HCC. The CIBERSORT algorithm was implemented to compare the infiltration proportions of immune cells between the two risk groups (Figure 10a). Higher ratios of M0 and M2 macrophages were observed in the high-risk group. In contrast, monocytes, CD8+ T cells and activated NK cells predominantly infiltrated the low-risk group. Most immune checkpoint genes (PDCD1, CD48, BTNL2, CD276, etc.) were remarkably upregulated in the high-risk group (Figure 10b). Single-sample gene set enrichment analysis (ssGSEA) was quantified to explore immune cell infiltration fraction and functions based on the m7GRLSig model. The immune cell score analysis presented significant differences in the infiltration fraction of aDCs, DCs, iDCs, macrophages, mast cells, Tfh cells, Th1/2 cells, and Tregs between the two groups (Figure 10c). The immune functions of APC co-stimulation, CCR, checkpoint, HLA, MHC class I, parainflammation, and T cell co-stimulation were upregulated in the high-risk group, whereas the type II IFN response was upregulated in the low-risk group (Figure 10d). These results elucidated the role of m7GRLSig in the immune landscape of HCC.

### 3.7. Correlations between Drug Treatment and m7GRLSig

**Figure 11:**
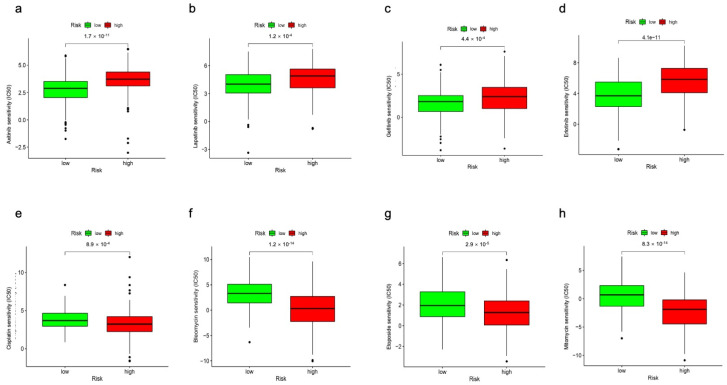
Comparison of treatment drugs sensitivity between high- and low-risk groups. The IC50 values for axitinib (**a**), lapatinib (**b**), gefitinib (**c**), erlotinib (**d**), cisplatin (**e**), bleomycin (**f**), etoposide (**g**), and mitomycin (**h**).

We further explored the correlations between the m7GRLSig risk score and the efficacy of drug treatment for HCC. Axitinib, lapatinib, gefitinib, and erlotinib were more sensitive in patients with a low m7GRLSig-score (Figure 11a–d), while cisplatin, bleomycin, etoposide, and mitomycin were more sensitive in patients with a high m7GRLSig-score (Figure 11e–h). The drug-m7GRLSig correlation analysis demonstrated that the m7GRLSig-score was related to the sensitivity to drugs and suggested that m7GRLSig was a potential predictor of HCC clinical treatment.

### 3.8. Knockdown of NRAV Inhibited Proliferation and Metastasis of HCC and Affected METTL1 Expression

**Figure 12:**
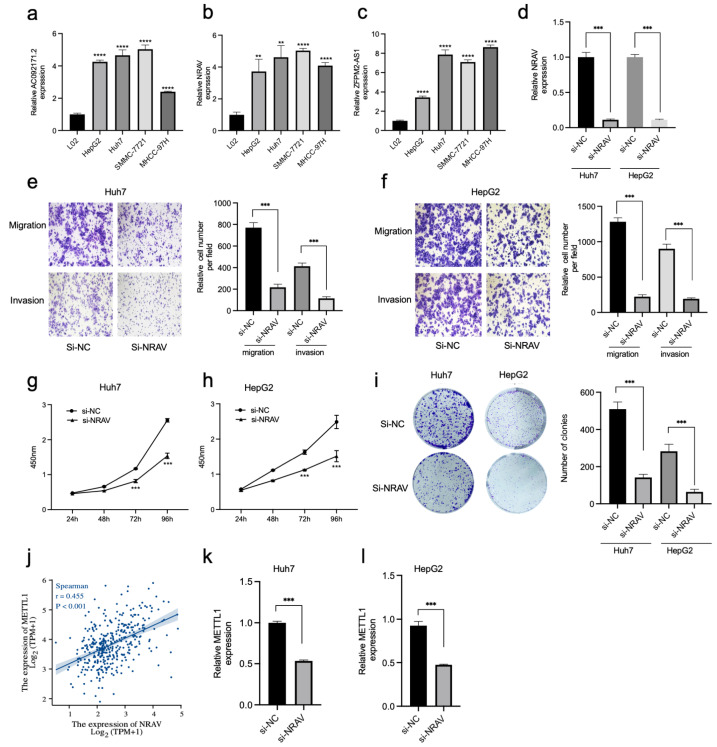
Knockdown of NRAV inhibited proliferation and metastasis of HCC and affected mettl1 expression. HCC cells and normal L02 cells were subjected to qRT-qPCR to verify the expression of AC092171.2 (**a**), NRAV (**b**), and ZFPM2-AS1 (**c**). mRNA expression of NRAV in Huh7 and HepG2 cells transfected with si-NRAV or si-NC (**d**). Transwell assays show the migration and invasion of Huh7 (**e**) and HepG2 (**f**) cells transfected with si-NRAV or si-NC. CCK-8 (**g**,**h**) and colony assays (**i**) of Huh7 and HepG2 cells transfected with si-NRAV or si-NC. (**j**) Correlation of NRAV and METTL1 in TCGA-LIHC database. Expression of NRAV mRNA in Huh7 (**k**) and HepG2 (**l**) cells transfected with si-NRAV or si-NC. HCC, hepatocellular carcinoma; NC, negative control; TCGA-LIHC, The Cancer Genome Atlas-Liver hepatocellular carcinoma. **, *p* < 0.01; ***, *p* < 0.001; ****, *p* < 0.0001.

PIK3CD-AS2, AC092171.2, NRAV, ZFPM2-AS1, and CASC19 were risk factors in the m7GRLSig model, as mentioned above; the expression of AC092171.2, NRAV, and ZFPM2-AS1 was significantly higher in tumor tissues in the TCGA cohort (Supplementary Figure S4a–e). The qRT-PCR analysis showed higher AC092171.2, NRAV, and ZFPM2-AS1 expression in HCC cell lines compared to L02 cell (Figure 12a–c). As shown in Figure 2c, most of these m7G-related genes were significantly associated with NRAV including METTL1, which was reported to promote HCC progression via m7G modification as the most critical regulator. Therefore, we evaluated the association between NRAV and METTL1 during HCC progression. NRAV mRNA levels were significantly decreased in cells treated with si-NRAV compared to the negative control (Figure 12d). Transwell assays revealed that the ability of migration and invasion of Huh7 and HepG2 cells were suppressed in si-NRAV pretreated cells compared to the negative control (Figure 12e,f). Furthermore, the knockdown of NRAV significantly decreased Huh7 and HepG2 cell proliferation compared to the negative control (Figure 12g–i). Given a strong positive correlation between NRAV and METTL1 (Figure 12j), we speculated that NRAV might also regulate the expression of METTL1 during HCC progression. The mRNA expression of METTL1 was remarkably downregulated in si-NRAV–pretreated Huh7 and HepG2 cells (Figure 12k,l). NRAV may positively regulate HCC progression by affecting METTL1 expression in vitro. These results indicated the prognostic lncRNAs in m7GRLSig may regulate the m7G modification during HCC progression by targeting the m7G regulator.

## 4. Discussion

The prognosis of patients with HCC is generally very poor because of the difficulties in early diagnosis. It is challenging to establish a robust HCC classification and develop effective therapies based on limited clinical characteristics due to high inter- and intratumor heterogeneity. To improve personalized diagnosis and the effectiveness of treatment, a growing number of studies have explored some potential molecular biomarkers that predict the diagnosis and prognosis of HCC patients.

To date, several studies reported that m7G modification promotes the development and progression of multiple cancers [29,30]. M7G modification of various RNAs serves a critical function in RNA processing and transcription [31]. The METTL1/WDR4 complex promotes hepatocarcinogenesis via m7G tRNA modification [14]. In addition, multiple studies have indicated that lncRNAs contribute to HCC progression. LncRNA W42 binds to DBN1 to promote HCC development [32]. LncRNA HULC modulates ferroptosis in HCC via directly binding the miR-3200-5p/ATF4 axis [33]. Lnc02154 facilitates HCC growth and metastasis likely via trigging the PI3K-AKT signaling [34]. However, the m7G-related lncRNAs in HCC ought to be explored.

Our research aimed to elucidate the expression pattern of m7G-related LncRNAs and their effects on the HCC prognosis. Seven m7G-related lncRNAs (ZFPM2-AS1, AC092171.2, PIK3CD-AS2, HPN-AS1, AC022613.1, NRAV, CASC19) were screened for a risk model to predict the HCC prognosis. As expected, patients with high risk had significantly worse outcomes compared with low-risk patients. Time-dependent ROC curves indicated the robustness of the risk model in predicting survival rate at 1, 3, and 5 years. Moreover, we constructed a nomogram integrating risk score and clinical features to predict HCC prognosis. The calibration curves at 1, 3, and 5 years showed that the actual survival corresponded well with the predicted survival. Our findings indicate that the potential prognostic value of this model is better than that of the traditional clinicopathological characteristics.

Some studies have identified that m7G-related genes or lncRNAs correlate with tumor microenvironment [35,36]. We linked m7GRLSig to the immune landscape of HCC. Immunological analysis showed that the m7GRLSig stratification was sensitive in the assessment of immunocytes infiltration proportion, immune checkpoint genes expression, immunocytes infiltration fraction and immune functions. The proportion of immune cells infiltrating contributes to the development of cancers [37]. We found that high-risk scores indicated higher infiltration of M0 and M2 macrophages, whereas higher infiltration of CD8^+^ T cells and activated NK cells were associated with a low-risk score based on our risk model. Consistent with the previous study, M2 macrophages play important roles in enhancing HCC growth and metastasis and CD8+ T cells are significantly associated with better HCC prognosis [38]. Meanwhile, the expression of most immune checkpoint genes (PDCD1, TIGIT, CD44, etc.) is highly associated with high-risk groups, which may identify patients who potentially benefit from immune checkpoint inhibitor (ICI) treatments. Targeting the m7G-related lncRNAs combined with checkpoint blockade may be a possible therapeutic strategy for HCC patients. The high-risk group showed a deteriorated immune status with a higher fraction of immune-suppressive cells (Tregs, tumor-associated macrophages [TAMs], Tfh cells). These results illustrated a significant relationship between m7G-related lncRNAs and the immune landscape of HCC. Taken together, these differences in the immune landscape between these two groups provide a potential therapeutic guide for immunotherapy due to the dependence of immunotherapy on a pre-existing immune microenvironment [37]. In addition, HCC is considered to be one of the most chemotherapy-resistant tumors [39]. Our research indicated that the high-risk group was sensitive to conventional treatments, including cisplatin, bleomycin, etoposide, and mitomycin. It is critical to predict the efficacy of different treatments on HCC patients. Taken together, this risk model could predict the sensitivity to HCC-related drugs and may contribute to personalized therapy for patients with HCC.

In previous reports, m6A modification and lncRNAs drive HCC through interactive regulation. The downregulation of RAB11B-AS1 caused by METTL16-induced m6A modification promotes HCC progression [40]. METTL3 increased the expression of LINC00958 via m6A modification during HCC progression [41]. ILF3-AS1 promoted the ILF3 m6A modification via recruiting METTL3 during HCC progression [42]. CASC11 promoted HCC progression by decreasing the m6A level of UBE2T with ALKBH5 recruiting [43]. LINC01273 decreased the METTL3 expression by enhancing the stability of miR-600 to confer sorafenib resistance [44]. To confirm our signature, we first detected the expression of these upregulated lncRNAs in cell lines. Consistent with expression levels of lncRNAs in TCGA, AC092171.2, NRAV, and ZFPM2-AS1 expression was upregulated in HCC cell lines compared to L02 cells. As most of these m7G-related genes are significantly associated with NRAV and METTL1 is the most critical regulator in m7G modification during HCC progression, we hypothesized that METTL1 and NRAV promote hepatocarcinogenesis through interactive regulation. We found that silencing NRAV suppressed the proliferation and metastasis of HCC and affected the mRNA expression levels of METTL1, whereas NRAV levels were not affected by METTL1 knockdown (data were not shown). These results revealed that the prognostic lncRNAs in m7GRLSig may regulate m7G modification by targeting the m7G regulator and provided new insights into the interactive regulation between lncRNA and m7G enzymes during HCC progression.

Several limitations of this risk model should be mentioned. First, the risk model was built based on the TCGA database; thus, independent cohorts should be used to validate the risk model. Second, this was a retrospective study. Lastly, the potential mechanisms of these lncRNAs with predictive value and their relationship with m7G modification should be clarified by further in-depth functional experiments.

In conclusion, our study provides evidence for a novel m7G-related lncRNA signature as a potential prognostic biomarker to independently predict the survival of HCC patients. This risk model may provide prospective therapeutic strategies and improve individualized drug treatments for HCC patients.

## Figures and Tables

**Table 1:** Multivariate Cox analysis screened 7 lncRNAs with prognostic significance.

LncRNAs	Coeffcient	HR	HR.95L	HR.95H
ZFPM2-AS1	0.181329605	1.198810248	0.946806641	1.517887549
AC092171.2	0.360712851	1.434351529	0.977113068	2.105553981
PIK3CD-AS2	0.340465024	1.405601077	0.932002427	2.119859704
HPN-AS1	−0.427741428	0.651979977	0.380372155	1.117531565
AC022613.1	−0.417716086	0.658549173	0.492770614	0.88009918
NRAV	0.488602295	1.630036318	0.929726329	2.85785001
CASC19	0.342542856	1.408524715	0.973281485	2.038405029

HR, hazard ratio; HR.95L low 95%CI of HR; HR.95H, high 95%CI of HR.

## Data Availability

Publicly available data sets were analyzed in this study. These data can be found here: https://portal.gdc.cancer.gov/, accessed on 6 May 2022.

## Supplementary Materials

The following supporting information can be downloaded at: https://www.mdpi.com/article/10.3390/curroncol30010035/s1, Figure S1: The Kaplan–Meier survival curves of seven prognostic M7Grelated lncRNAs; Figure S2: Verification of m7G-related lncRNAs prognostic signature’s prediction ability and risk score analysis in entire set; Figure S3: Kaplan–Meier survival analysis for subgroups with different clinicopathological features; Figure S4: The expression of PIK3CD-AS2, AC092171.2, NRAV, ZFPM2-AS1, and CASC19 in tumor and normal tissues in TCGA; Table S1: Clinical pathological parameters of patients with HCC; Table S2: Primers and their sequence; Table S3: Differential expression analysis 29 M7G-related genes in HCC and normal tissue; Table S4: Univariate Cox regression screened 84 lncRNAs with prognostic significance.
