# Supplementary material for: Identification of a Novel N7-Methylguanosine-Related LncRNA Signature Predicts the Prognosis of Hepatocellular Carcinoma and Experiment Verification
Source: Curr Oncol. 2022 Dec 28;30(1):430–48. doi: 10.3390/curroncol30010035 (PMC9857529; doi:10.3390/curroncol30010035)
Supplement: Supplementary file 1 [file curroncol-30-00035-s001.zip › curroncol-2098897-supplementary.pdf]

Identification of a Novel N7-Methylguanosine-Related LncRNA Signature Predicts the Prognosis of Hepatocellular Carcinoma and Experiment Verification

Chou Yang †, Lingyan Zhang †, Xin Hao, Mengdie Tang, Bin Zhou * and Jinlin Hou *

State Key Laboratory of Organ Failure Research, Guangdong Key Laboratory of Viral Hepatitis Research, Department of Infectious Diseases and Hepatology Unit, Nanfang Hospital, Southern Medical University, Guangzhou 510515, China
* Correspondence: ice1126@163.com (B.Z.); jlhousmu@163.com (J.H.)
† These authors have contributed equally to this work and share first authorship.

Supplementary tables

Table S1. Clinical pathological parameters of patients with HCC

characteristics		N	percent
Status	Alive	241	64.96
	Dead	130	35.04
Age	Mean (SD)	59.4 (13.5)	
	Median [Min, Max]	61 [16,90]	
Gender	Female	121	32.61
	Male	250	67.39
T classification	T1	181	48.79
	T2	92	24.80
	T2a	1	0.27
	T2b	1	0.27
	T3	45	12.13
	T3a	29	7.82
	T3b	6	1.62
	T4	13	3.50
	TX	1	0.27
N classification	N0	252	67.92
	N1	4	1.08
	NX	114	30.73

M classification	M0	266	71.70
	M1	4	1.08
	MX	101	27.22
TNM stage	I	171	46.09
	II	86	23.18
	III	3	0.81
	IIIA	65	17.52
	IIIB	8	2.16
	IIIC	9	2.43
	IV	2	0.54
	IVA	1	0.27
	IVB	2	0.54
Grade	G1	55	14.82
	G2	177	47.71
	G3	122	32.88
	G4	12	3.23

SD, Standard Deviation; T, tumor size; N, lymph node metastasis; M, distant metastasis.

Table S2. Primers and their sequence

Primers	sequence (5′-3′)
METTL1(forward)	GGCAACGTGCTCACTCCAA
METTL1(reverse)	CACAGCCTATGTCTGCAAACT
AC092171.2(forward)	CAAAAGCCTTGCGGAGTAGA
AC092171.2(reverse)	ACAATTACGGCGTCTCGGA
NRAV(forward)	GGAGTTGATGCCTCCGAACA
NRAV(reverse)	ATGACCGGAGCTGAAAGGTG
ZFPM2-AS1(forward)	CAATGGGACTAAGCCAGGCA
ZFPM2-AS1(reverse)	GGGCTCCACCAACAACCATA

GAPDH (forward)	CTCACCGGATGCACCAATGTT
GAPDH(reverse)	CGCGTTGCTCACAATGTTCAT

Table S3. Differential expression analysis 29 M7G-related genes in HCC and normal tissue.

genes	conMean	treatMean	logFC	*P* value
METTL1	2.51141954	5.683383818	1.17824709	4.50E-19
WDR4	1.083637722	3.269096707	1.59300954	7.59E-25
NSUN2	7.10043896	10.44918126	0.55740978	3.25E-12
DCP2	1.153536534	2.265097875	0.97350969	3.42E-17
DCPS	8.87724002	13.49670393	0.60442401	7.33E-02
NUDT10	0.305789794	0.06591064	-2.213957	1.70E-24
NUDT11	0.016429097	0.183072781	3.47809224	2.87E-03
NUDT16	6.94674804	9.089878516	0.38792324	6.59E-05
NUDT3	1.08906101	1.86192756	0.77371217	1.11E-15
NUDT4	1.733881698	2.443665803	0.49504153	1.12E-04
NUDT4B	0.000553711	0.00190915	1.78572459	2.74E-01
AGO2	0.575442362	1.885179913	1.71195888	3.15E-23
CYFIP1	5.00382744	7.580696179	0.59929831	1.59E-10
EIF4E	0.840025222	1.041636307	0.31034709	4.12E-03
EIF4E1B	0.000736658	0.002805457	1.92916853	3.77E-01
EIF4E2	5.71196626	9.039683717	0.66228484	1.30E-18
EIF4E3	1.288465648	0.904269866	-0.5108288	4.62E-06
GEMIN5	1.716665852	3.075102333	0.84102517	1.22E-15
LARP1	7.79047786	19.54303962	1.32687114	1.82E-24
NCBP1	3.2891083	4.464767594	0.44088857	5.07E-06
NCBP2	4.0674739	8.706112187	1.09789552	4.66E-23
NCBP3	0.994576602	1.379807231	0.47231233	3.88E-05
EIF3D	16.5830878	37.20819698	1.16590782	1.78E-22

EIF4A1	0.364938682	0.579985625	0.66836306	1.19E-04
EIF4G3	2.609384278	5.064699826	0.95676734	2.89E-13
IFIT5	3.95744964	4.473113047	0.17670823	1.38E-01
LSM1	4.83314204	7.358223912	0.60639618	5.79E-07
NCBP2L	0.026472469	0.046489641	0.81241652	9.80E-01
SNUPN	2.08851324	3.636030214	0.7998879	1.10E-22

Table S4. Univariate Cox regression screened 84 lncRNAs with prognostic significance.

lncRNAs	HR	HR.95L	HR.95H	*P* value
BACE1-AS	1.223601	1.106203	1.353458	8.81E-05
AC068473.5	1.442546	1.198912	1.73569	1.04E-04
AC099850.3	1.152714	1.099602	1.208392	3.52E-09
AC020915.3	1.369186	1.11014	1.688679	3.32E-03
AL117336.3	1.625027	1.343457	1.96561	5.70E-07
AL050341.2	1.171015	1.061043	1.292385	1.70E-03
ZFPM2-AS1	1.096951	1.055428	1.140109	2.60E-06
LINC02362	0.933434	0.875058	0.995705	3.66E-02
AL390198.1	1.103958	1.016963	1.198394	1.82E-02
ZNF529-AS1	1.290573	1.022131	1.629516	3.20E-02
AC068580.1	1.213141	1.021714	1.440435	2.74E-02
LINC00623	1.242552	1.0677	1.446039	5.01E-03
AC092171.2	1.085548	1.018441	1.157076	1.17E-02
AC084033.3	1.104888	1.009435	1.209367	3.05E-02
FOXD2-AS1	1.17224	1.078007	1.274711	2.02E-04
GASAL1	1.418948	1.155472	1.742502	8.41E-04
PRRT3-AS1	1.09782	1.043206	1.155294	3.38E-04
AL606489.1	1.21591	1.052161	1.405144	8.07E-03
AC115619.1	0.981002	0.969826	0.992306	1.03E-03

PIK3CD-AS2	1.261489	1.152777	1.380454	4.37E-07
AC096536.2	1.456666	1.08699	1.952065	1.18E-02
AL365203.2	1.149741	1.087051	1.216046	1.07E-06
TMEM220-AS1	0.738642	0.630658	0.865116	1.72E-04
MIR210HG	1.159298	1.082971	1.241005	2.10E-05
AC008549.1	0.981368	0.967219	0.995725	1.11E-02
LINC01138	1.498439	1.260886	1.780747	4.39E-06
AP001065.1	0.900482	0.829849	0.977128	1.19E-02
SNHG16	1.102999	1.004574	1.211068	3.98E-02
LINC00261	0.98286	0.968772	0.997154	1.89E-02
AL354892.2	1.103552	1.010941	1.204647	2.76E-02
AC100847.1	1.290491	1.054351	1.57952	1.34E-02
ZEB1-AS1	1.471319	1.176976	1.839274	6.97E-04
AC010761.1	1.439121	1.139613	1.817345	2.23E-03
AC016747.1	1.18116	1.067335	1.307125	1.28E-03
SNHG20	1.269978	1.050166	1.5358	1.37E-02
NRSN2-AS1	1.280838	1.080381	1.518489	4.37E-03
AL049840.4	1.070045	1.02136	1.121051	4.38E-03
OTUD6B-AS1	1.116845	1.016504	1.22709	2.14E-02
AC005332.5	1.18888	1.062809	1.329907	2.49E-03
AC040970.1	1.221185	1.083803	1.375982	1.03E-03
HPN-AS1	0.763691	0.591484	0.986035	3.87E-02
BX537318.1	1.231714	1.03263	1.46918	2.05E-02
SBF2-AS1	1.435463	1.135104	1.8153	2.54E-03
LYRM4-AS1	1.393703	1.104519	1.758601	5.15E-03
AC009005.1	1.186448	1.087147	1.294818	1.26E-04
F11-AS1	0.856652	0.763769	0.960832	8.23E-03
AC074117.1	1.446159	1.165124	1.794982	8.19E-04
AC022613.1	0.943469	0.896458	0.992945	2.56E-02

AC009403.1	1.176459	1.081085	1.280246	1.65E-04
AC010969.2	1.343624	1.08768	1.659794	6.15E-03
AC006504.8	1.387486	1.142022	1.685709	9.78E-04
SNHG3	1.073115	1.042481	1.104649	1.79E-06
MAPKAPK5-AS1	1.064507	1.005001	1.127537	3.32E-02
AC145207.5	1.834458	1.409276	2.387919	6.48E-06
PTOV1-AS1	1.254261	1.052136	1.495215	1.15E-02
NRAV	1.250558	1.142511	1.368823	1.24E-06
SREBF2-AS1	1.498501	1.19707	1.875833	4.16E-04
NIFK-AS1	1.284039	1.057725	1.558776	1.15E-02
AL355574.1	1.276445	1.100784	1.480138	1.23E-03
DANCR	1.018681	1.004095	1.033479	1.19E-02
SNHG12	1.140094	1.038616	1.251485	5.84E-03
WAC-AS1	1.091984	1.044489	1.141639	1.05E-04
AC124798.1	1.24092	1.111282	1.385681	1.26E-04
AC026401.3	1.095107	1.046615	1.145846	8.44E-05
AC009779.2	1.186951	1.093101	1.288858	4.54E-05
PCAT6	1.088416	1.012132	1.170449	2.23E-02
AL359504.2	1.46259	1.102827	1.939713	8.30E-03
CASC19	1.157224	1.073774	1.24716	1.31E-04
AC004816.1	1.21965	1.08485	1.3712	8.91E-04
FGD5-AS1	1.028182	1.00358	1.053387	2.45E-02
AC073611.1	1.44347	1.128709	1.846009	3.45E-03
Z95115.1	1.231329	1.067435	1.420388	4.30E-03
AC006026.3	1.382073	1.106381	1.726463	4.36E-03
AC012146.1	1.077697	1.004679	1.156023	3.66E-02
AC093673.1	1.030448	1.001811	1.059904	3.70E-02
AC004656.1	1.151622	1.078386	1.229833	2.54E-05
MIR4435-2HG	1.127349	1.045675	1.215403	1.78E-03

AL031985.3	1.897344	1.547871	2.325719	7.00E-10
AL355488.1	1.155009	1.012349	1.317772	3.22E-02
SNHG7	1.030315	1.004721	1.056561	2.00E-02
AC015908.3	0.662815	0.547668	0.802171	2.40E-05
RAB11B-AS1	0.968486	0.938011	0.999951	4.96E-02
AC145343.1	1.307122	1.085737	1.573646	4.67E-03
CYTOR	1.035646	1.011731	1.060126	3.30E-03

HR, hazard ratio; HR.95L low 95%CI of HR; HR.95H, high 95%CI of HR

Supplementary Figures

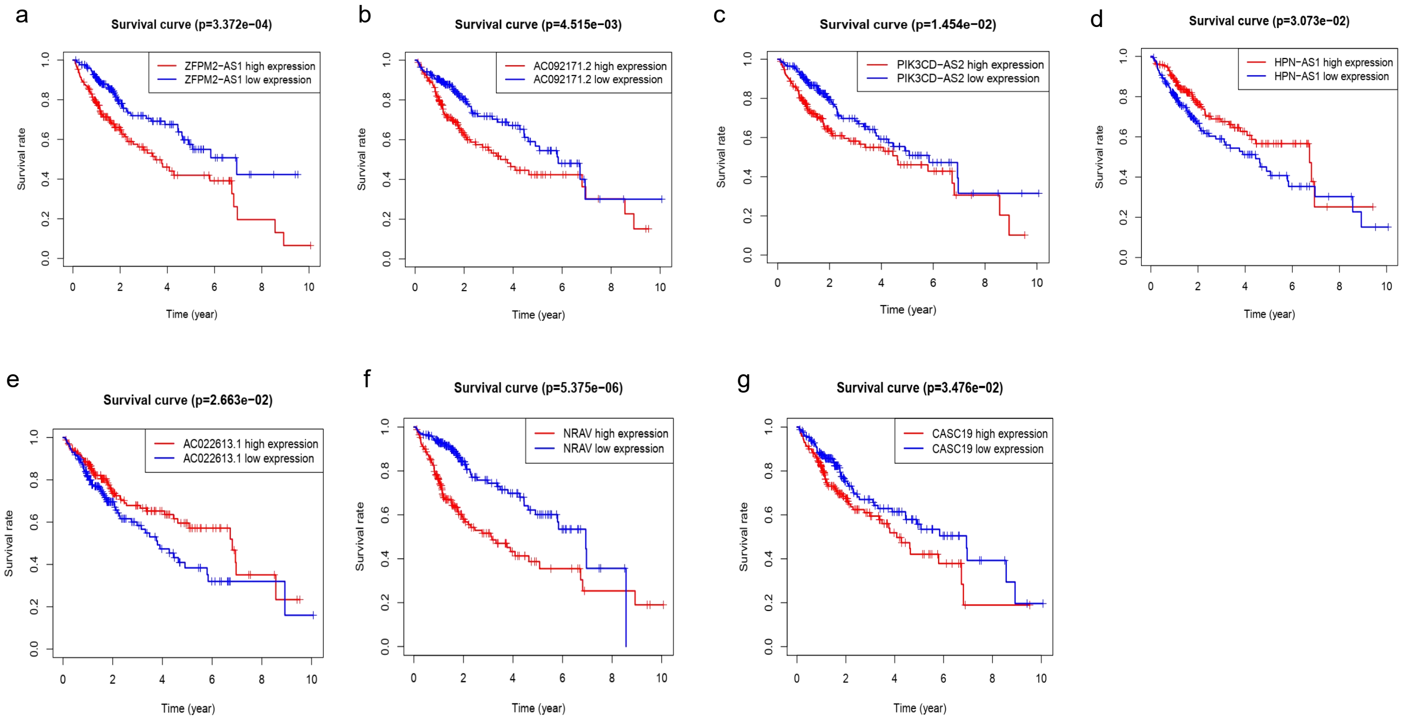

Figure S1. The Kaplan–Meier survival curves of seven prognostic M7G-related lncRNAs.

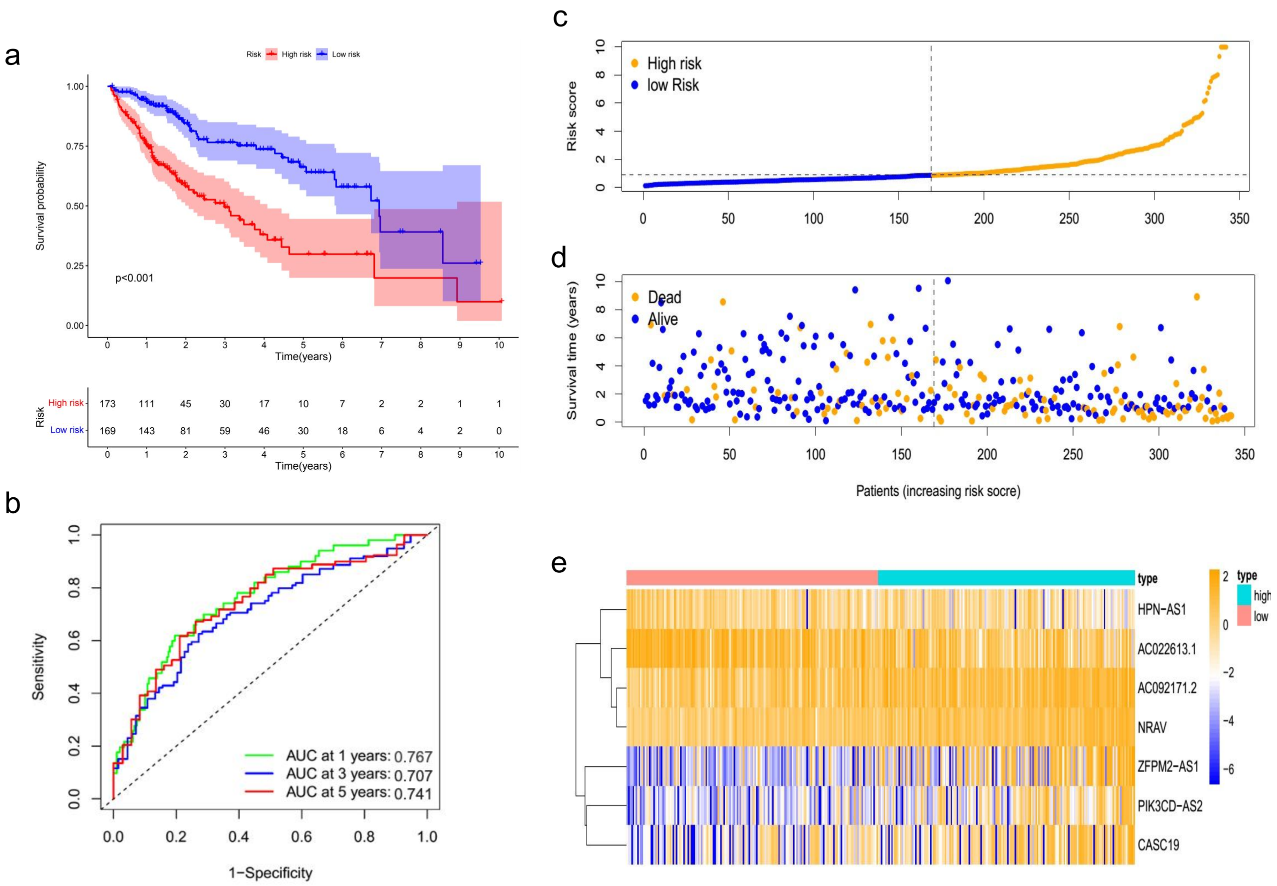

Figure S2. Verification of m7G-related lncRNAs prognostic signature's prediction ability and risk score analysis in entire set.

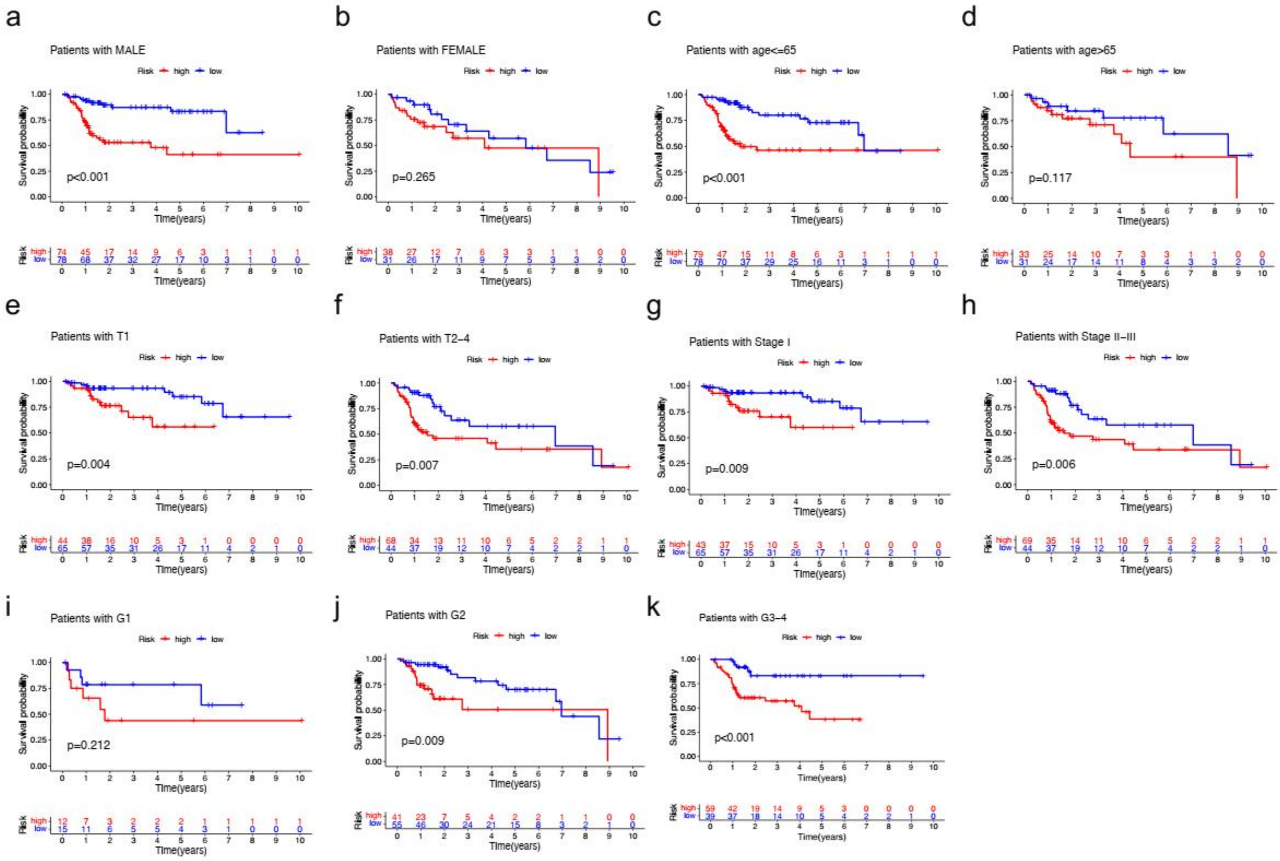

Figure S3. Kaplan–Meier survival analysis for subgroups with different clinicopathological features.

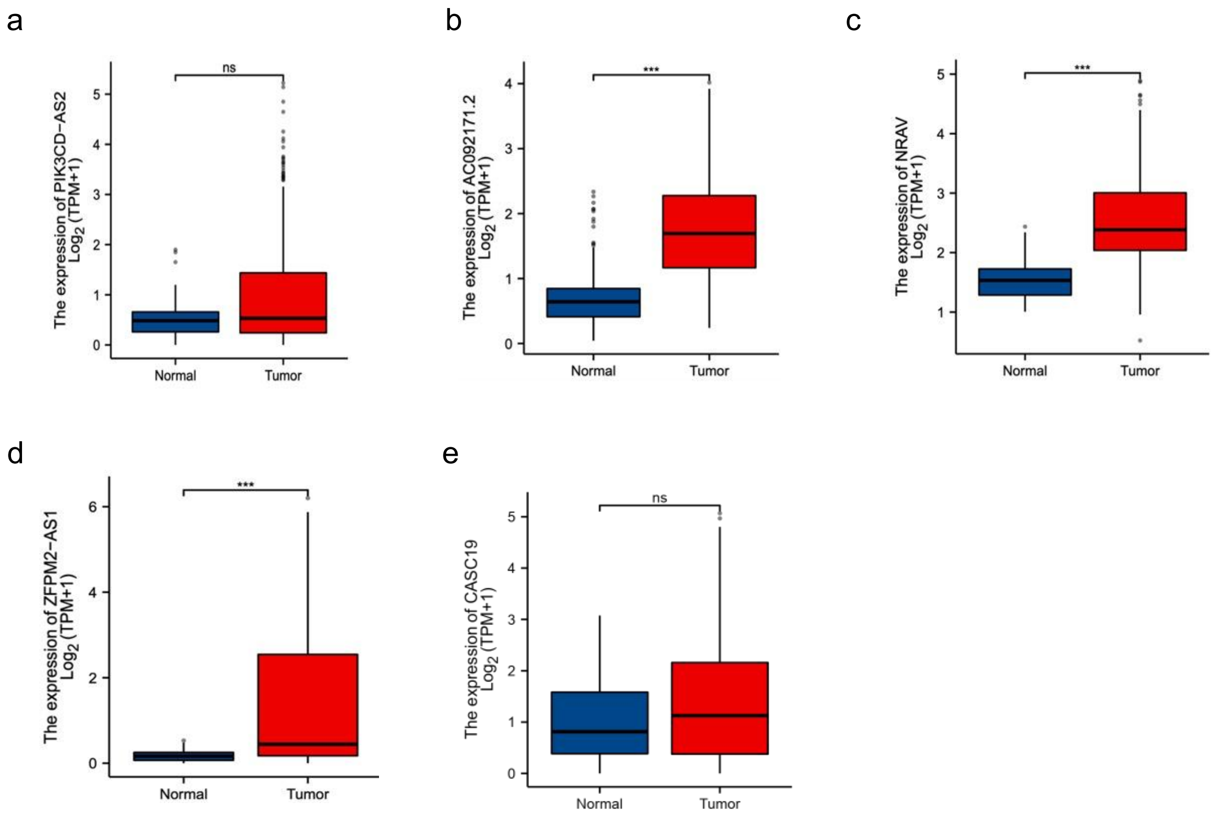

Figure S4. The expression of PIK3CD-AS2, AC092171.2, NRAV, ZFPM2-AS1, and CASC19 in tumor and normal tissues in TCGA.
